# Improved differentiation between primary lung cancer and pulmonary metastasis by combining dual-energy CT–derived biomarkers with conventional CT attenuation

**DOI:** 10.1007/s00330-020-07195-9

**Published:** 2020-08-28

**Authors:** Dominik Deniffel, Andreas Sauter, Alexander Fingerle, Ernst J. Rummeny, Marcus R. Makowski, Daniela Pfeiffer

**Affiliations:** 1 Department of Radiology, Klinikum rechts der Isar, School of Medicine, Technical University of Munich, Ismaninger Str. 22, 81675 Munich, Germany; 2 Lunenfeld-Tanenbaum Research Institute, Sinai Health System, University of Toronto, Toronto, ON Canada

**Keywords:** Neoplasms lung, Tomography X-ray computed, Lung cancer, Contrast media

## Abstract

**Objectives:**

To assess the clinical utility of dual-energy CT (DE-CT)–derived iodine concentration (IC) and effective Z (Z^eff^) in addition to conventional CT attenuation (HU) for the discrimination between primary lung cancer (LC) and pulmonary metastases (PM) from different primary malignancies.

**Methods:**

DE-CT scans of 79 patients with LC (3 histopathologic subgroups) and 89 patients with PM (5 histopathologic subgroups) were evaluated. Quantitative IC, Z^eff^, and conventional HU values were extracted and normalized to the thoracic aorta. Differences between groups were assessed by pairwise Welch’s *t* test. Correlation and linear regression analyses were used to examine the relationship of imaging parameters in LC and PM. Diagnostic accuracy was measured by the area under receiver operator characteristic curve (AUC) and validated based on resampling methods.

**Results:**

Significant differences between subgroups of LC and PMs were noted for all imaging parameters, with the highest number of significant pairs for IC. In univariate analysis, only IC was a significant diagnostic feature for discriminating LC from PM (*p* = 0.03). All quantitative imaging parameters correlated significantly (*p* < 0.0001, respectively), with the highest correlation between IC and Z^eff^ (*r* = 0.91), followed by IC and HU (*r* = 0.76) and Z^eff^ and HU (*r* = 0.73). Diagnostic models combining IC or Z^eff^ with HU (IC+HU: AUC = 0.73; Z^eff^+HU: AUC = 0.69; IC+Z^eff^+HU: AUC = 0.73) were not significantly different and outperformed individual parameters (IC: AUC = 0.57; Z^eff^: AUC = 0.57; HU: AUC = 0.55) in diagnostic accuracy (*p* < 0.05, respectively).

**Conclusion:**

DE-CT-derived IC or Z^eff^ and conventional HU represent complementary imaging parameters, which, if used in combination, may improve the differentiation between LC and PM.

**Key Points:**

*• Individual quantitative imaging parameters derived from DE-CT (iodine concentration, effective Z) and conventional CT (HU) provide complementary diagnostic information for the differentiation of primary lung cancer and pulmonary metastases.*

*• A combination of conventional HU and DE-CT parameters enhances the diagnostic utility of individual parameters.*

**Electronic supplementary material:**

The online version of this article (10.1007/s00330-020-07195-9) contains supplementary material, which is available to authorized users.

## Introduction

Computed tomography (CT) represents the cornerstone modality for the imaging of pulmonary malignancies. Lung cancer (LC) is the most common cancer in the USA and causes the most cancer-related deaths per organ site [1]. However, the most common malignant tumor found in the lungs are pulmonary metastases (PM) originating from another primary cancer [2]. The differentiation between primary LC and PM is critical, as it defines the diagnostic and therapeutic workflow. For example, diagnosis of PM usually warrants further imaging studies to identify the primary malignancy (e.g., mammography and other organ-specific imaging studies), whereas LC requires complete staging examinations before definitive treatment can be initiated. Additionally, patients may present with synchronous PM and primary LC [3] or with a known primary tumor and a new pulmonary lesion of unknown origin (PM or LC). Until recently, upon appearance of an unknown pulmonary lesion on CT, radiologists used morphological criteria, such as density, borders, and size, to narrow down the list of possible differential diagnoses [4, 5]. These criteria are particularly useful for the differentiation between malignant and benign pulmonary lesions. However, in most cases, further subclassification of lesions is not possible based on conventional CT alone, ultimately requiring biopsy or follow-up examinations for definite diagnosis.

Over the last years, dual-energy CT (DE-CT) has emerged as a promising diagnostic technology for various clinical applications [6–10] leading to widespread adoption in clinical practice. Previous studies exploring the use of DE-CT for the assessment of pulmonary lesions focused on the differentiation between lung cancer (LC) and inflammatory masses [11] on the one hand and the differentiation of PMs [12, 13] on the other hand. To our knowledge, no previous studies have investigated the differentiation of LC and PM by conventional CT or DE-CT, despite the given clinical relevance. In previous DE-CT studies, the most frequently used imaging parameter was iodine concentration (IC). More recently, the effective atomic number (Z^eff^) decomposition of tissues was introduced for oncologic applications. Similar to IC, the rationale of using Z^eff^ is accurate quantification of iodine uptake, considered a surrogate measure for tumor perfusion. Both imaging parameters are now readily available in commercial DE-CT analysis packages. This, however, has raised a further dilemma for radiologists in clinical practice: which quantitative imaging parameters should be used for the characterization of pulmonary lesions or can they be used interchangeably? Furthermore, would conventional Hounsfield units (HU) still provide an incremental diagnostic value or is this parameter obsolete when IC or Z^eff^ measurements are available? Only few studies assessed all of the latter imaging parameters simultaneously and for pulmonary malignancies, no formal analysis has been presented as to which quantitative imaging parameters is to be preferred.

In this study, we sought to systematically assess the diagnostic value of IC and Z^eff^, derived from DL-CT, as well as conventional HU for the characterization of numerous pulmonary malignancies. We hypothesized that incorporating IC, Z^eff^, and HU into a diagnostic model may improve the differentiation between pulmonary metastases and LC and thus help streamline the diagnostic workflow in patients with pulmonary lesions of unknown origin.

## Material and methods

### Patient population

**Fig. 1:**
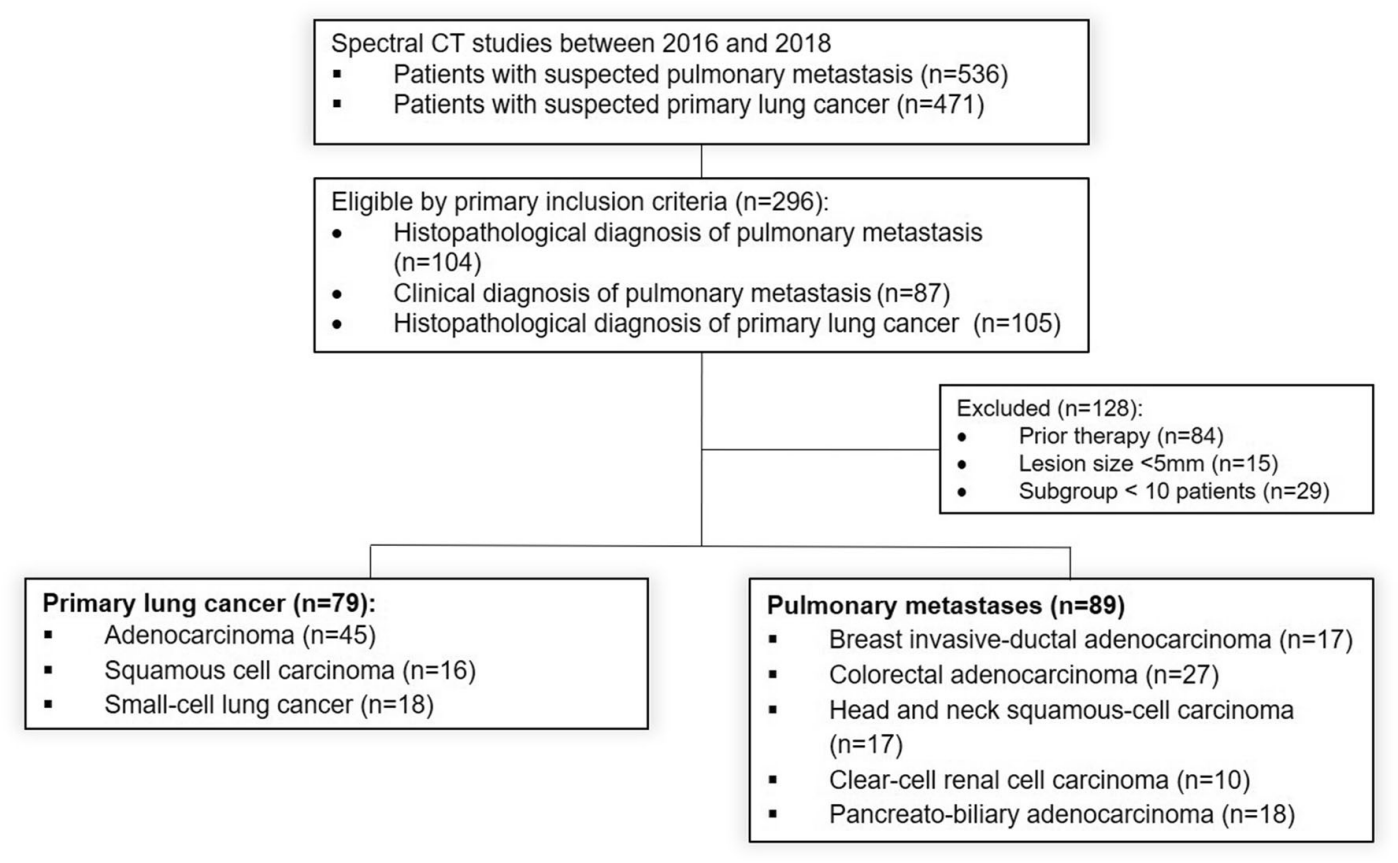
Patient selection process

This retrospective, HIPAA-compliant, single-center study was approved by our institutional review board, and a waiver of informed consent was obtained. Between 2016 and 2018, 1007 patients underwent a DL-CT of the thorax for suspected PM or suspected primary lung tumors. Primary inclusion criteria were (a) histopathologically confirmed primary LC, (b) histopathologically confirmed PM, and (c) clinically confirmed PM. Clinical diagnosis of PM was based on a follow-up > 6 months demonstrating progression of the PM, defined by the appearance of new lesions or > 20% progression in size. Histopathological subgroups smaller than 10 patients were excluded from further analysis. Furthermore, patients were excluded due to prior therapy (local, systemic) and lesion size less than 5 mm. We finally analyzed 79 patients with primary LC (adenocarcinoma, *n* = 45; squamous cell carcinoma (SCC), *n* = 16; small-cell LC (SCLC), *n* = 18) and 89 patients with PM from primary breast (invasive-ductal adenocarcinoma, *n* = 17), colorectal (CRC) (adenocarcinoma, *n* = 27), head and neck (squamous cell carcinoma, *n* = 17), kidney (RCC) (clear-cell renal cell carcinoma, *n* = 10), and pancreato-biliary (PBC) (adenocarcinoma, *n* = 18) malignancies. The study population flowchart is illustrated in Fig. 1.

### Dual-energy CT technique

The examination of all patients using a dual-layer spectral CT (IQon; Philips Healthcare) followed the same routine protocol. Before the start of the scan, an anterior-posterior scout was performed to determine the scan range. Intravenous contrast medium (Imeron 400 MCT, 400 mg/ml; Bracco Imaging) was injected with a standard dosage of 80 ml at a flow rate of 2–2.5 ml/s, followed by a 30-ml saline chaser at the same flow rate. All scans were performed in the venous phase (scan delay 70 s after the start of the application of the contrast medium). The following scanning parameters were used: 120 kVp; automatic tube current selection with resulting exposures of 37–84 mAs; 512 × 512 matrix; collimation 64 × 0.625 mm; reconstructed slice thickness and interval 0.9 mm/0.9 mm with a soft tissue kernel. The field-of-view was adapted to the patient size. Conventional and spectral basis images were reconstructed using the iDose^4^ (Philips Healthcare) algorithm. The mean volume-weighted CT dose index (CTDI_vol_) and dose length product (DLP) for the complete protocol were 4.4 mGy and 180 mGy*cm, respectively, corresponding to an effective dose of 2.5 mSv (conversion factor 0.014).

### Image analysis

Quantitative analysis of spectral CT images was performed using commercially available software (IntelliSpace Portal v. 10.1, Philips Healthcare). Image analysis was performed retrospectively by a resident fellow in radiology and supervised by a senior radiologist (11 years of radiology experience). Using a semi-automated 3D segmentation algorithm, a volume-of-interest was created for each pulmonary lesion. A circular two-dimensional region-of-interest was manually placed in the descending aorta. The following imaging parameters were acquired: conventional (polychromatic) Hounsfield units (HU), iodine concentration (IC) (mg/ml), and effective atomic number (Z^eff^). To account for hemodynamic inter-patient variations, the measured parameter values in the volume-of-interest of the PM were normalized to the thoracic aorta as previously described [13]. All analyses were conducted on normalized measurements.

### Statistical analysis

Reporting followed Standards of Reporting of Diagnostic Accuracy [14]. All statistical analyses were performed using R version 3.6.1 (R Foundation for Statistical Computing) and the following R packages: caret (versions 6.0–84), boot (versions 1.3–22), car (versions 3.0–3), pROC (1.15.3) [15]. Confidence intervals for all analyses were derived using bootstrap sampling with 2000 replicates. Differences between means were assessed by pairwise Welch’s *t* test to correct for unequal variances between groups. Multiple testing adjusted *p* values were computed using Benjamini-Hochberg correction [16], also known as false-discovery rate. Differences were only assessed between subgroups of metastases and LC. The relationship between imaging parameters was evaluated using the Pearson correlation coefficient (*r*) and linear regression analysis. Three-parameter linear regression models were constructed using pairs of imaging parameters as independent and dependent variable, the type of pulmonary tumor as categorical variable (primary versus metastatic), and two-way interactions between them. This approach allows to assess the change of one imaging parameter value in a tumor subgroup as a function of another imaging parameter. Logistic regression models were developed for the discrimination of primary LC versus PM using imaging parameters alone and in combination. Bootstrapping with 2000 replications was performed for internal validation to avoid optimism in model performance measures. Diagnostic accuracy was measured by the area under receiver operator characteristic (ROC) curve (AUC) and compared using a bootstrap test (2000 replicates) [15]. Additional diagnostic performance measures (sensitivity, specificity, and negative and positive predictive value) were calculated for model thresholds corresponding to the maximum Youden index. All tests were two-tailed, and *p* < 0.05 was considered statistically significant.

## Results

**Fig. 2:**
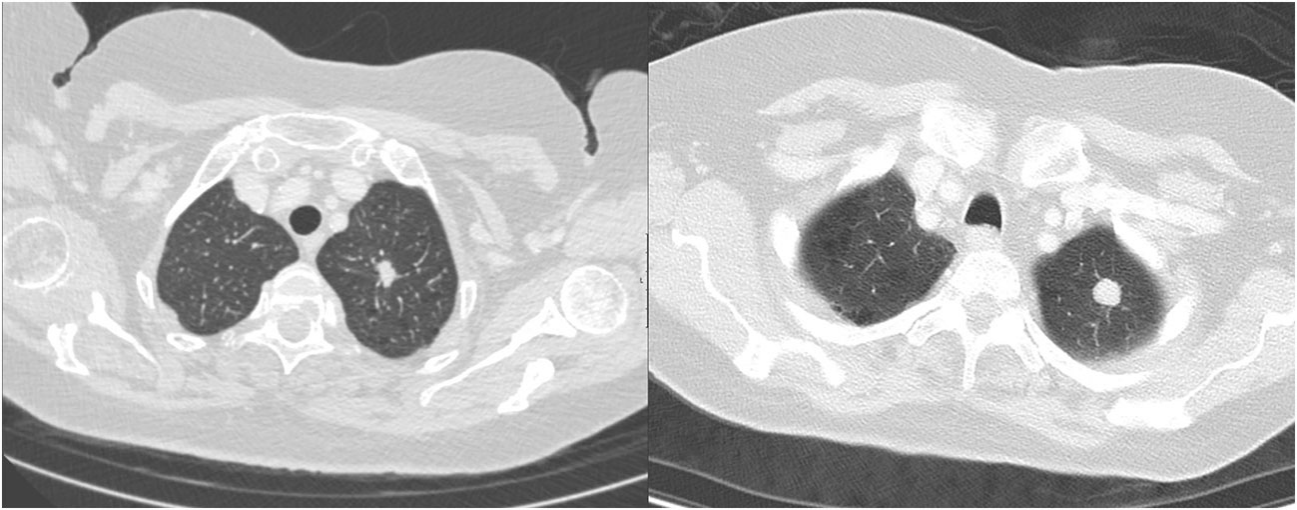
Examples of a patient with primary lung cancer (left) and with pulmonary metastasis (right). In each patient, a lesion in the apex of the left upper lobe is present. The following values were measured for these lesions: HU: 61/68; Z^eff^: 8.4/7.3; IC 1.84/0.60

**Fig. 3:**
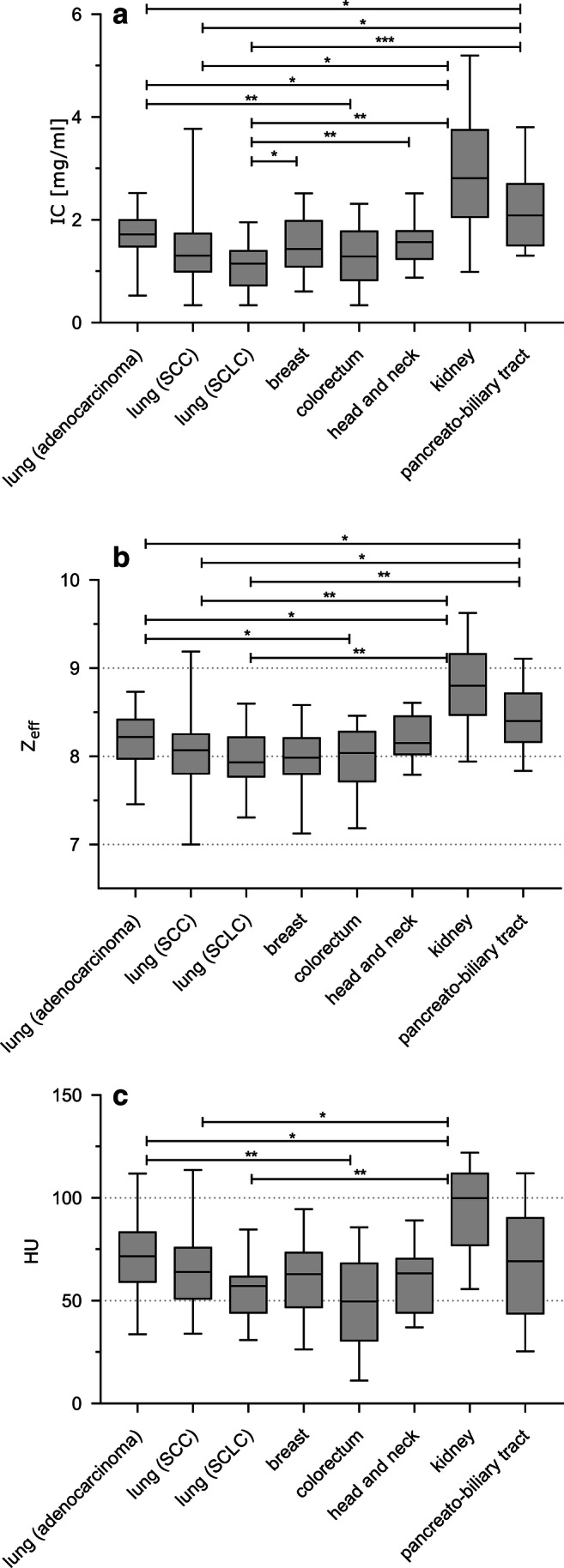
Quantitative results for the dual-energy CT–derived iodine concentration and conventional CT attenuation values. **a** Median values of iodine concentration (IC), (**b**) effective atomic number (Z^eff^), and (**c**) conventional Hounsfield unit (HU) values with quartiles, minimum, and maximum values, for primary lung cancers (adenocarcinoma, squamous cell carcinoma (SCC), small-cell lung cancer (SCLC)) and pulmonary metastases from primary breast (invasive-ductal adenocarcinoma), colorectal (adenocarcinoma), head and neck (squamous cell carcinoma), kidney (clear-cell renal cell carcinoma), and pancreato-biliary (adenocarcinoma) malignancies. Presented are *p* values of pairwise Welch *t* tests adjusted for multiple comparisons using the Benjamini-Hochberg correction. Differences between subgroups of pulmonary metastases and primary lung cancers were not assessed. *Adjusted *p* value of < 0.05, ** < 0.01, *** < 0.001

A total of 79 LC and 89 PM on 168 DE-CT scans were analyzed in this study; a representative example of a LC and a PM is shown in Fig. 2. Quantitative results of IC, Z^eff^, and conventional CT values of all pulmonary tumors are provided in Table 1 and Fig. 3a–c. Significant differences in all imaging parameters were observed for lung adenocarcinoma versus PM from CRC (IC: *p* = 0.01; Z^eff^: *p* = 0.05; HU: *p* = 0.002) and RCC (IC: *p* = 0.02; Z^eff^: *p* = 0.01; HU: *p* = 0.04), for SCC versus PM from RCC (IC: *p* = 0.02; Z^eff^: *p* = 0.005; HU: *p* = 0.02), and for SCLC versus PM from RCC (IC: *p* = 0.009; Z^eff^: *p* = 0.003; HU: *p* = 0.002). Additional significant differences were noted based on IC and Z^eff^ between lung adenocarcinoma (IC: *p* = 0.02; Z^eff^: *p* = 0.02), SCC (IC: *p* = 0.02; Z^eff^: *p* = 0.02), and SCLC (IC: *p* = 0.0001; Z^eff^: *p* = 0.003) versus metastatic lesions from PBC, respectively. Significant differences between SCLC and metastases from head and neck cancer (IC: *p* = 0.01) and breast cancer (IC: *p* = 0.04) were only observed for IC.

**Table 1:** Iodine concentration, effective Z, conventional HU, and tumor volume of lung metastases and primary lung cancers

Type	Primary location	Histology	*n*	IC (mg/ml)	Z^eff^	HU
Metastasis	Breast	Invasive-ductal adenocarcioma	17	1.5 ± 0.59	8.00 ± 0.37	60.39 ± 18.95
Metastasis	Colorectum	Adenocarcioma	27	1.26 ± 0.56	7.97 ± 0.38	50.5 ± 22.34
Metastasis	Head and neck	Squamos cell carcinoma	17	1.57 ± 0.46	8.21 ± 0.27	59.72 ± 15.89
Metastasis	Kidney	Clear-cell carcinoma	10	2.89 ± 1.24	8.81 ± 0.51	94.39 ± 22.77
Metastasis	Pancreato-biliary tract	Adenocarcioma	18	2.2 ± 0.76	8.43 ± 0.35	67.73 ± 25.62
Primary tumor	Lung	Adenocarcioma	45	1.66 ± 0.47	8.18 ± 0.21	71.10 ± 17.1
Primary tumor	Lung	Squamos cell carcinoma	16	1.44 ± 0.78	8.04 ± 0.46	65.98 ± 20.78
Primary tumor	Lung	Small-cell lung cancer	18	1.09 ± 0.42	7.96 ± 0.39	55.53 ± 14.2

Listed are mean values ± standard deviation. Pulmonary tumors are specified by their type (primary, metastatic), location of their primary tumor, and histology. The following parameters were measured using dual-energy CT: *IC*, iodine concentration; *Z*^*eff*^, effective atomic number; *HU*, conventional attenuation values in Hounsfield units

**Fig. 4:**
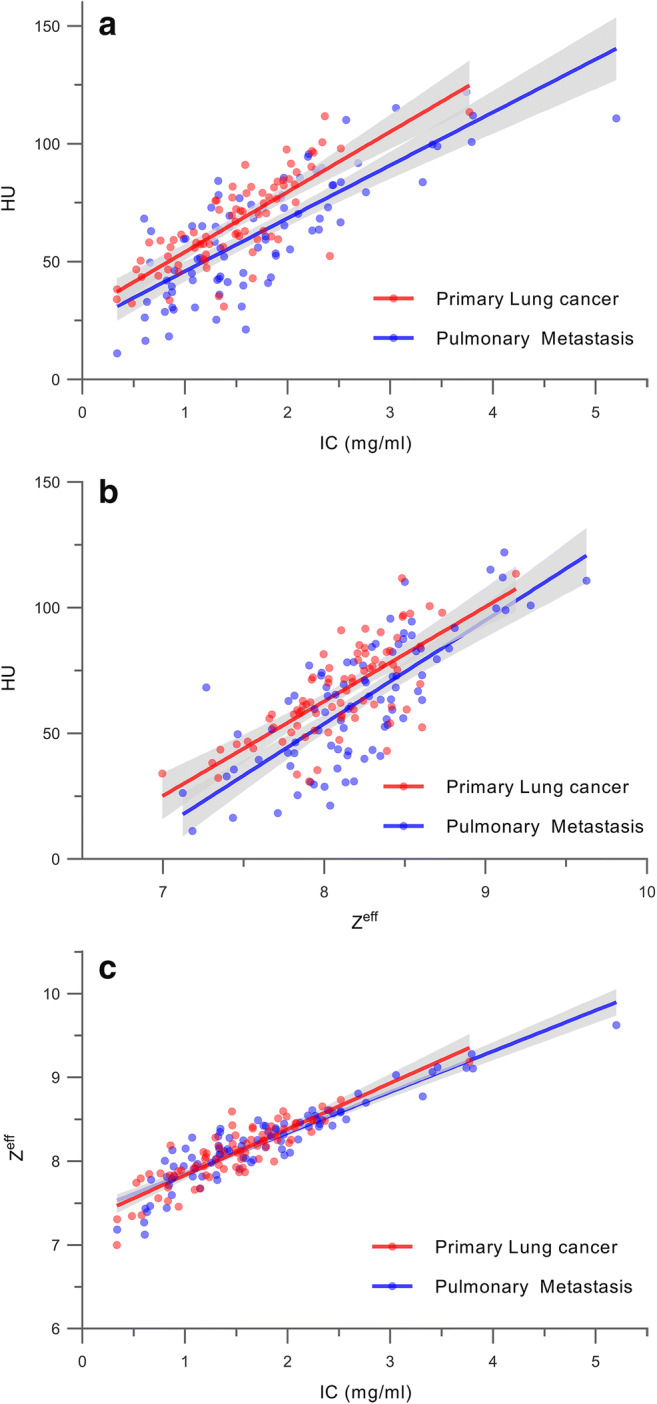
Linear regression scatter plots of quantitative imaging parameters in primary lung cancer and pulmonary metastases. **a** Conventional HU and IC. **b** Conventional HU and Z^eff^. **c** Z^eff^ and IC. Gray-shaded area represents the limits of the 95% confidence interval of the linear regression fit. Significant differences in HUs were noted between primary lung cancer and pulmonary metastases at fixed IC (*p* < 0.0001) and Z^eff^ (*p* = 0.0003) in regression models **a** and **b**; no significant difference in Z^eff^ at fixed IC was found in model **c**

All quantitative imaging parameters correlated significantly, with the highest correlation between IC and Z^eff^ (*r* = 0.91, *p* < 0.0001), followed by IC and HU (*r* = 0.76, *p* < 0.0001) and Z^eff^ and HU (*r* = 0.73, *p* < 0.0001). We analyzed the following linear regression models including the type of pulmonary tumor (primary versus metastatic) and pairs of imaging parameter as dependent versus independent variable (Table 2): model (a) HU versus IC (Fig. 4a); model (b) HU versus Z^eff^ (Fig. 4b); model (c) Z^eff^ versus IC (Fig. 4c). A two-way interaction term was not statistically significant in any of the three regression models (model a: *p* = 0.94; model b: *p* = 0.53; model c: *p* = 0.13) and was, therefore, dropped from the models. A significant effect of the type of pulmonary tumor on HU values (dependent variable) was observed for models a (*p* < 0.0001) and b (*p* < 0.0001), with coefficients of 9.85 and 8.43, respectively, for the category “Primary LC.ˮ These results translate into significantly higher HU values of LCs compared with those of PM at fixed values of IC (model a) and Z^eff^ (model b), and, by contrast, no significant differences in Z^eff^ at fixed values of IC (model c).

**Table 2:** Linear regression analysis

Model	Dependent variable	Independent variables	Coeffecient [CI]	*p* value
a	HU	IC	23.36 [19.64, 26.49]	*< 0.0001*
		Primary lung cancer	9.85 [5.4, 13.85]	*< 0.0001*
		Pulmonary metastasis	Reference	
b	HU	Z^eff^	39.9 [34.12, 44.72]	*< 0.0001*
		Primary lung cancer	8.43 [3.63, 12.62]	*0.0003*
		Pulmonary metastasis	Reference	
c	Z^eff^	IC	0.50 [0.46, 0.55]	*< 0.0001*
		Primary lung cancer	0.02 [− 0.04, 0.07]	0.59
		Pulmonary metastasis	Reference	

CI indicates bootstrapped 95% confidence interval (2000 replicates). *IC*, iodine concentration on dual-energy computed tomography; *HU*, conventional computed tomography attenuation values in Hounsfield units; *Z*^*eff*^, effective atomic number. Italics indicate significant *p* values (< 0.05). Note that two-way interaction terms for all three models were not significant in preliminary analyses

**Fig. 5:**
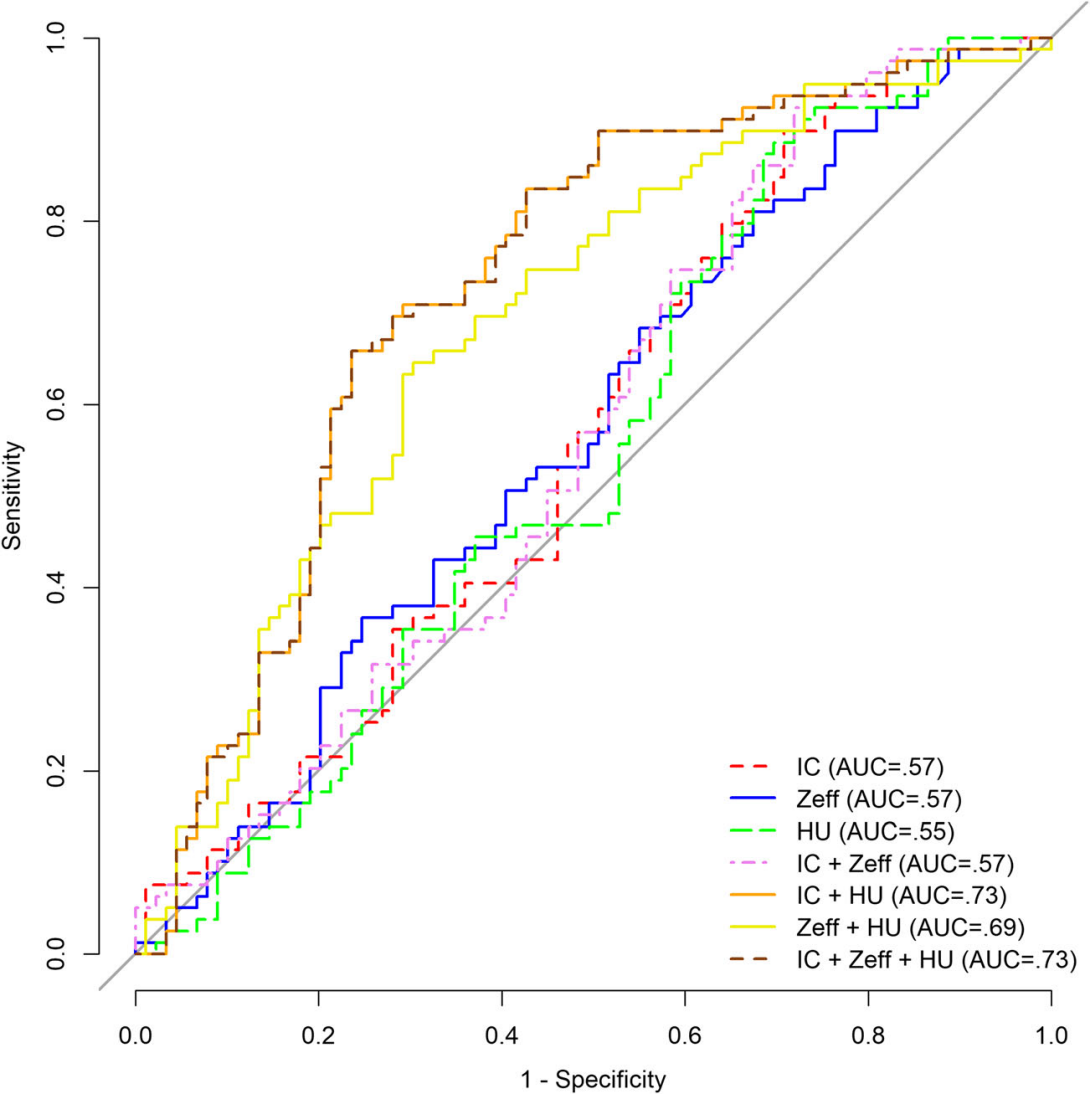
ROC curves for the discrimination of primary lung cancer (*n* = 79) from pulmonary metastases (*n* = 89) corrected for optimism using bootstrapping (2000 replicates). Logistic regression models, combining dual-energy CT–derived iodine concentration (IC), Z^eff^, and conventional CT attenuation values (HU), as well as single parameters, were tested. The corresponding areas under the curve (AUCs) of the combined models incorporating HU were significantly higher than those of IC (*p* < 0.05), Z^eff^ (*p* < 0.01), or HU (*p* < 0.01) alone. No significant differences were noted between single parameters and a combined model using IC and Z^eff^

In univariate logistic regression analysis, only IC was found to be a significant predictor for the discrimination of primary LC from PM (*p* = 0.03; Table 3). Coefficients of uni- and multivariate logistic regression models are provided in Supplementary Table 1. Diagnostic performance was assessed for all imaging parameters alone and in combination (Table 4; Supplementary Table 2). Only the corresponding AUCs of logistic regression models combining HU with IC and/or Z^eff^ were significantly different from 0.5 (*p* < 0.0001, respectively), indicating useful diagnostic tests. Accordingly, the corresponding bootstrap optimism-corrected AUCs of these combinations were significantly higher compared with those of single imaging parameters and a combination of IC and Z^eff^ (*p* < 0.005, respectively). The combined models including HU (IC+HU, Z^eff^+HU, IC+Z^eff^+HU) did not differ significantly in their corresponding AUCs. The highest AUC was achieved by the parameter combination of IC and HU (AUC 0.73) (Table 4; Fig. 5).

**Table 3:** Univariate logistic regression analysis for discrimination of primary lung cancer from pulmonary metastases

Variable	Coeffecient	[CI]	*p* value
IC	− 0.481	[− 0.95, − 0.07]	*0.03*
Z^eff^	− 0.66	[− 1.36, 0.13]	0.09
HU	0.01	[− 0.01, 0.02]	0.25

CI indicates bootstrapped 95% confidence intervals (2000 replicates). *IC*, iodine concentration on dual-energy computed tomography; *HU*, conventional computed tomography attenuation values in Hounsfield units; *Z*^*eff*^, effective atomic number. Italic indicates significant *p* values (< 0.05)

**Table 4:** Diagnostic accuracy for the differentiation of primary lung cancer from pulmonary metastases

Model	AUC [CI]	*p*^a^	*p*^b^	*p*^c^	*p*^d^	*p*^e^	*p*^f^	*p*^g^
IC	0.57 [0.48, 0.65]		0.95	0.83	*0.0009*	*0.02*	1	*0.001*
Z^eff^	0.57 [0.48, 0.66]	0.95		0.82	*0.002*	*0.01*	0.96	*0.002*
HU	0.55 [0.46, 0.63]	0.83	0.82		*0.0003*	*0.006*	0.83	*0.0003*
IC+HU	0.73* [0.65, 0.80]	*0.0009*	*0.002*	*0.0003*		0.17	*0.0009*	*0.36*
Z^eff^+HU	0.69* [0.61, 0.77]	*0.02*	*0.01*	*0.006*	0.17		*0.04*	*0.17*
IC+Z^eff^	0.57 [0.48, 0.65]	*1*	0.96	0.83	*0.0009*	*0.04*		*0.0009*
IC+Z^eff^+HU	0.73* [0.65, 0.80]	*0.001*	*0.002*	*0.0003*	0.36	0.17	*0.0009*	

*AUC*, area under the receiver operating characteristic curve; *CI*, bootstrapped 95% confidence intervals (2000 replicates); *IC*, iodine concentration on dual-energy computed tomography; *HU*, conventional computed tomography attenuation values in Hounsfield units; *Z*^*eff*^, effective atomic number. *p* values are provided for the comparisons of bootstrap optimism-corrected AUCs: versus ^a^IC; ^b^Z^eff^; ^c^HU; ^d^IC+HU; ^e^Z^eff^+HU; ^f^IC+Z^eff^; ^g^IC+Z^eff^+HU. Italicized *p* values indicate significant differences (< 0.05). *AUCs significantly different from 0.5

## Discussion

Our study demonstrates that the discrimination of primary LC from PM is feasible using a combination of DE-CT-derived IC or Z^eff^, and conventional HU. Using either imaging parameter alone did not enable diagnostic utility. IC and Z^eff^ did not provide complementary information to one another for this diagnostic task and can thus be used interchangeably for the implementation into a diagnostic model.

In earlier reports, DE-CT-derived parameters were used for the differentiation of PM from different primary tumors [12, 13] and for the evaluation of therapy response in LC [17]. However, to our knowledge, no previous studies have examined the differentiation between LC and PM. For the majority of cases in current clinical practice, this discrimination can only be made based on histopathological evaluation. Therefore, patients are required to undergo biopsy upon appearance of new pulmonary lesions on CT. Aside from possible complications, such as pneumothorax or bleeding, this diagnostic pathway may ultimately delay further therapy. Our study suggests DE-CT as a useful tool for the differentiation of LC and PM, which may help streamline the diagnostic workflow in patients with pulmonary lesions of unknown origin and even avoid invasive diagnostic procedures in certain cases.

Our study focused on the utility of the most commonly used DE-CT-derived parameters IC and Z^eff^, which are readily provided by most commercial software platforms, in addition to conventional HU. While previous DE-CT studies used a multitude of imaging features, such as the slope of the spectral HU curve [18], virtual mono-energetic HUs, or extracted radiomic features from DE-CT-derived parametric maps [19], the appeal of our approach is its simplicity, only using three “off-the-shelfˮ imaging parameters obtained from DE-CT scans which could facilitate integration into the clinical workflow without additional software applications.

Our study could demonstrate that a differentiation between LC and PM is possible in many cases. However, if used alone, no parameter provided reliable predictions. Both IC and Z^eff^ are considered surrogate measures for tumor vascularity and perfusion. While IC directly quantifies iodine content, Z^eff^ describes the average atomic number for a tissue of interest and can thus indirectly provide information about accumulation of contrast material. Coefficients for IC and Z^eff^ in the logistic regression models indicate that LC accumulated less contrast material than PMs, indicating differences in tumor vascularity. Nonetheless, diagnostic utility could only be achieved by the addition of HU in a diagnostic model. This diagnostic incremental value of HU was confirmed by linear regression analysis. At fixed values of IC or Z^eff^, LCs demonstrated significantly higher HU values than PMs. This suggests that, in addition to tumor vascularity, which may be accurately assessed by IC or Z^eff^, there are subtle differences in tissue composition that are more evident by HU quantification. Consequently, the assessment of pulmonary tumors of unknown origin in clinical routine should not be limited to single parameters but consider both conventional CT attenuation and DE-CT-derived parameters.

Not all pairwise comparisons between subgroups of LC and PM in our study yielded significant differences for all imaging parameters. Given the similarity of several subgroups of LC and PM, in many cases, a definite differentiation may not be achieved by imaging alone. In a clinical context, our proposed combined diagnostic models could, however, provide an additional diagnostic hint and help streamline further diagnostic work-up. For instance, upon appearance of a new pulmonary lesion in patients with a known primary tumor, characteristic DE-CT parameter values may help to confirm the clinical suspicion and initiate therapy without additional invasive tissue sampling.

Findings of our study should be interpreted in the context of some limitations. First, the analysis was retrospective and limited to a single reference center. Second, the number of included CT scans for some histopathological subgroups was relatively low and, although we assessed a multitude of tumor types, no benign pulmonary lesions and not all malignant pulmonary tumors are considered. Third, no other DE-CT technology was used besides DL-CT as different systems are not available at our institution. Since IC can be measured accurately with other systems [20], the results of the present study are expected to be transferable to other systems. Fourth, we acknowledge that additional imaging characteristics such as tumor margins, size, calcifications, or presence of necrotic areas, which are an integral part of any clinical CT assessment, could have improved the diagnostic performance of our model. This study is a first step in demonstrating the feasibility of distinguishing between LC and PM. Machine learning algorithms handling multiple predefined input variables or deep learning models that are capable of automatically learning and extracting imaging features are an interesting avenue for future research but will ultimately require larger data sets.

In conclusion, our study demonstrates the feasibility of differentiating between LC and PM via DE-CT. Using a combination of IC and/or Z^eff^ with HU may help avoid invasive tissue sampling in patients with a history of extra- or intrapulmonary malignancies.

## Electronic supplementary material

ESM 1 (DOCX 21 kb)

## Abbreviations

AUC: Area under the receiver operating characteristic curve
CRC: Colorectal adenocarcinoma
CT: Computed tomography
CTDI_vol_: Volume-based CT dose index
DE-CT: Dual-energy CT
DL-CT: Dual-layer CT
DLP: Dose length product
DS-CT: Dual-source CT
HU: Hounsfield units
IC: Iodine concentration
LC: Lung cancer
PBC: Pancreato-biliary adenocarcinoma
PM: Pulmonary metastasis
RCC: Renal cell carcinoma
ROC: Receiver operating characteristic
ROI: Region-of-interest
SCC: Squamous cell carcinoma
SCLC: Small-cell lung cancer
SD: Standard deviation
SEM: Standard error of the mean
