# Supplementary material for: Improved differentiation between primary lung cancer and pulmonary metastasis by combining dual-energy CT–derived biomarkers with conventional CT attenuation
Source: Eur Radiol. 2020 Aug 28;31(2):1002–10. doi: 10.1007/s00330-020-07195-9 (PMC7813728; doi:10.1007/s00330-020-07195-9)
Supplement: Supplementary file 1 — (DOCX 21 kb) [file 330_2020_7195_MOESM1_ESM.docx]

**Electronic Supplementary Material**

**Supplementary Table 1.** Logistic regression models for the discrimination of primary lung cancer from pulmonary metastases

| Model | Variable | Coefficient | [95% CI] | p-value |
| --- | --- | --- | --- | --- |
| ‘IC’ | (Intercept) | 0.65 | [-0.11, 1.46] | 0.1 |
|  | IC | -0.48 | [-0.95, -0.07] | 0.03 |
| ‘Z^eff^‘ | (Intercept) | 5.28 | [-1.13, 11.05] | 0.09 |
|  | Z^eff^ | -0.66 | [-1.36, 0.13] | 0.09 |
| ‘HU’ | (Intercept) | -0.65 | [-1.55, 0.32] | 0.18 |
|  | HU | 0.01 | [-0.01, 0.02] | 0.25 |
| ‘IC+HU’ | (Intercept) | -0.77 | [-1.68, 0.33] | 0.15 |
|  | IC | -1.85 | [-2.87, -0.97] | <0.0001 |
|  | HU | 0.06 | [0.03, 0.09] | <0.0001 |
| ‘Z^eff^+HU’ | (Intercept) | 15.75 | [5.8, 25.57] | 0.0005 |
|  | Z^eff^ | -2.27 | [-3.62, -0.89] | 0.0003 |
|  | HU | 0.04 | [0.01, 0.07] | 0.0005 |
| ‘IC+Z^eff^’ | (Intercept) | -3.12 | [-16.84, 9.53] | 0.63 |
|  | IC | -0.75 | [-1.82, 0.21] | 0.14 |
|  | Z^eff^ | 0.52 | [-1.2, 2.46] | 0.56 |
| ‘IC+Z^eff^+HU’ | (Intercept) | -0.36 | [-16.03, 13.36] | 0.96 |
|  | IC | -1.82 | [-3.1, -0.61] | 0.003 |
|  | Z^eff^ | -0.06 | [-1.97, 2.12] | 0.95 |
|  | HU | 0.06 | [0.03, 0.09] | <0.0001 |

CI indicates bootstrapped 95% confidence intervals (2000 replicates); IC, iodine concentration on dual-energy computed tomography; HU, conventional computed tomography attenuation values in Hounsfield units; Z^eff^, effective atomic number.

**Supplementary Table 2.** Diagnostic performance of logistic regression models for the discrimination of primary lung cancer from pulmonary metastases.

| Model | Threshold* | % Sensitivity | % Specificity | % NPV | % PPV |
| --- | --- | --- | --- | --- | --- |
| ‘IC’ | 0.41 | 90 [44, 99] | 29 [16, 76] | 76 [60, 95] | 53 [50, 62] |
| ‘Z^eff^’ | 0.43 | 81 [33, 99] | 33 [13, 83] | 66 [57, 91] | 52 [50, 68] |
| ‘HU’ | 0.43 | 89 [51, 100] | 30 [16, 71] | 75 [61, 100] | 53 [50, 60] |
| ‘IC+HU’ | 0.53 | 66 [61, 94] | 76 [47, 84] | 72 [69, 90] | 71 [60, 79] |
| ‘Z^eff^+HU’ | 0.51 | 65 [51, 90] | 70 [43, 84] | 69 [64, 85] | 65 [57, 76] |
| ‘IC+Z^eff^’ | 0.39 | 94 [68, 100] | 27 [17, 56] | 83 [64, 100] | 53 [51, 60] |
| ‘IC+Z^eff^+HU’ | 0.53 | 66 [61, 94] | 76 [47, 83] | 72 [69, 91] | 71 [60, 79] |

*Determined by the maximum Youden index. Bootstrapped 95% confidence intervals (2000 replicates) in brackets. IC indicates iodine concentration on dual-energy computed tomography; HU, conventional computed tomography attenuation values in Hounsfield units; NPV, negative predictive value; PPV, positive predictive value; Z^eff^, effective atomic number.
